# 3D Convex Hull-Based Registration Method for Point Cloud Watermark Extraction

**DOI:** 10.3390/s19153268

**Published:** 2019-07-25

**Authors:** Bogdan Lipuš, Borut Žalik

**Affiliations:** Faculty of Electrical Engineering and Computer Science, University of Maribor, Koroška Cesta 46, SI-2000 Maribor, Slovenia

**Keywords:** point cloud registration, multi-scale registration, point cloud alignment, point cloud watermarking, remote sensing

## Abstract

Most 3D point cloud watermarking techniques apply Principal Component Analysis (PCA) to protect the watermark against affine transformation attacks. Unfortunately, they fail in the case of cropping and random point removal attacks. In this work, an alternative approach is proposed that solves these issues efficiently. A point cloud registration technique is developed, based on a 3D convex hull. The scale and the initial rigid affine transformation between the watermarked and the original point cloud can be estimated in this way to obtain a coarse point cloud registration. An iterative closest point algorithm is performed after that to align the attacked watermarked point cloud to the original one completely. The watermark can then be extracted from the watermarked point cloud easily. The extensive experiments confirmed that the proposed approach resists the affine transformation, cropping, random point removal, and various combinations of these attacks. The most dangerous is an attack with noise that can be handled only to some extent. However, this issue is common to the other state-of-the-art approaches.

## 1. Introduction

Presently, digital data are exchanging through the networks intensively, and are, in this way, exposed to various attacks. A small piece of additional information, i.e., a watermark, can be embedded within the data to identify their origin. Different approaches for watermarking of various digital multimedia data types, including images, audio, and video, were proposed in the past [1,2,3]. The watermark can be embedded in the spatial (for example, changing the grey levels of some pixels on the image) or frequency domains, where different transformations are applied, such as, for example, the discrete cosine or discrete wavelet transformations. The obtained coefficients of the transformed data can then be watermarked in the latest case. With the wide accessibility of 3D scanning devices [4,5], a huge amount of discrete points (i.e.,point clouds), acquired from the surfaces of 3D objects, are currently obtained easily. Their applicability is in various areas, such as, for example, cultural heritage [6,7,8], civil engineering [9,10], architecture [11,12,13], mechanical engineering [14,15], 3D simulation and animation [16,17,18], and indoor navigation [19,20,21]. There is, therefore, also a need to watermark the point cloud data. Unfortunately, the actual point cloud watermarking methods do not address all possible watermark attacks adequately. For example, because they use Principal Component Analysis (PCA) [22], they cannot protect the watermarked point clouds against cropping and random removal attacks.

A novel approach for point cloud watermarking is presented in this paper. It uses a new registration procedure, based on a 3D convex hull, applied before extraction of the watermark. This registration method determines the proper scale and alignment between the watermarked (possibly cropped and/or otherwise attacked) and the original point cloud. Consequently, the Principal Component Analysis (PCA) is not needed during the watermark process anymore.

The paper is organised as follows: Section 2 overviews related works. Section 3 introduces the proposed approach. The experimental results are given and discussed in Section 4. The paper is concluded in Section 5.

## 2. Previous and Related Works

Despite the wide applicability of point clouds, their watermarking has not been studied intensively. Principal Component Analysis (PCA) has been applied mostly to withstand affine transformation attacks [3,22,23]. Cotting et al. obtained surface patches by a fast hierarchical clustering algorithm and PCA [24]. After that, the surface patches are transformed into discrete frequency bands by applying the approximate Laplacian operator. The watermark was then embedded into their low-frequency components. Wang et al. also applied the PCA [25]. The points in the transformed coordinate system are then sorted for each axis to form intervals, which embed positions. The watermark was embedded by changing the point coordinates using a secret key. Ke et al. employed an octree and PCA to partition a 3D surface into patches [26]. Then, a patch chain was built to perform a similarity measure between them. The watermark was embedded into the average local vector of every similar patch. Agarwal and Prabhakaran considered these PCA-based approaches, and figured out that they are very vulnerable to cropping attacks [27]. Thus, they developed a new approach that constructs a cluster-tree by using the nearest neighbour heuristic. They applied an extended 3D quantization index modulation [28] to embed a watermark inside a cluster that consisted of at least three points: An encoding point, a head point, and a reference point. Unfortunately, their method depends on the mutual relations and the distances between the cluster points. If any point that defines the cluster, is removed or, for example, the point is not correctly identified as the header point, the watermark information cannot be extracted from that cluster. Therefore, Agarwal and Prabhakaran admitted that their method is not robust against simplification and cropping attacks. Luo et al. constructed clusters of eight 3D points around a randomly chosen vertex [29]. The coordinates of the points in each cluster were then used as an input to the Discrete Cosine Transformation (DCT) [30]. A watermark was inserted in the last DCT coefficients. Modified coefficients were then transformed inversely into coordinates in the spatial domain. Their method is vulnerable to any cluster modifications, such as a rearrangement of the points, and a point removal attack. However, the authors did consider how their method handles affine transformations and cropping attacks. As their method constructs the cluster of the closest points around a randomly selected vertex, it is unlikely that the clusters can be preserved when a random point removal and cropping attacks occur. Moreover, it is not clear how arbitrarily chosen vertexes are selected identically at a point removal and cropping attacks. They did not apply any registration of both point clouds. Thus, the affine transformations are not supported. Rames et al. presented a fragile fractal scheme for watermarking of LiDAR data, which can handle a huge number of points [31]. Their method incorporates computationally intensive operations and is not robust to attacks. The approach applies a General-Purpose Graphics Processing Unit (GPGPU) that was used to find similar fractal patterns. Recently, another fragile data hiding scheme was proposed by Itier and Puech [32]. Unfortunately, it has low robustness against attacks that can change the way of a Hamiltonian path that was constructed and used in data hiding. Table 1 summarises the level of robustness against various attacks.

Alternative approaches have been considered, because the PCA-based approaches cannot handle cropping and random removal attacks. The Iterative Closest Point algorithm (ICP) and its variants are registration techniques that could estimate rigid transformation, such as a translation and a rotation between the source and the target point cloud [33,34,35]. However, it is still a challenging task to estimate a non-rigid transformation (the simplest among them is scaling). A comprehensive survey on these topics can be found in [36]. Moreover, the ICP algorithms depend on a good introductory alignment to achieve accurate convergence to the global minimum. Thus, various approaches were proposed for the initial coarse alignment [37,38,39,40,41]. Unfortunately, these approaches still have difficulties with non-rigid transformation estimation, such as scaling. Only a few recent works consider this type of transformation. Mellado et. al. proposed a relative scale estimation and 3D registration of multi-modal geometry using growing least square that can handle situations with 3D models with different sampling densities, scale, and various levels of noise [42]. Their method supports both a semi-automatic scale estimation (the user needs to specify a scale range and a pair of corresponding points), and an automatic approach by selecting seeds and finding correspondences between them. In the final stage, a random sample consensus (RANSAC) is applied to estimate the relative scale and initial alignment [43]. Recently, Fan et al. proposed a registration approach that applies a 3D convex hull [44]. They tried to find the similarity transformation by determining a translation vector, a rotation matrix and a scaling factor. The best transformation is determined by a randomly selected triangle from the source convex hull and a triangle on the target convex hull by applying RANSAC. The same authors extended their work, and projected the cloud points onto the reference plane and then performed matching [45]. None of the above methods are used in the watermarking of a 3D point cloud. Thus, we are not convinced that they can handle different attacks properly, especially the cropping attacks. A more robust approach is needed that can handle the point clouds damaged by various attacks. Thus, we propose an improved registration method used in the process of watermark extraction, which also applies a 3D convex hull to determine the initial scaling factor between the watermarked (possibly attacked) and the original point clouds.

## 3. The Proposed 3D Point Registration Method

The main contribution of this paper is a new registration method for a relative scale estimation using a 3D convex hull in the process of the watermark extraction from a point cloud [46,47]. The main motivation was to overcome the main disadvantage of methods using the RANSAC algorithm and its variants. Namely, RANSAC is an iterative algorithm, operating on all points from the point cloud. The points are unstructured and, therefore, difficult for the registration. The number of geometric entities were reduced and the structured entity (i.e., the 3D convex hull) is obtained, which is more suitable for the registration. The proposed approach is an extension of our method for watermarking of georeferenced airborne LiDAR data [48], which does not consider the affine transformation attacks, because these types of attacks are meaningless for LiDAR data. On the contrary, the presented extension works on point clouds in general, and can handle affine transformation, cropping, and random removal attacks. In the continuation, a brief overview of the method for watermarking georeferenced airborne LiDAR data is given in the next subsection [48]. After that, the new registration method for watermark extraction from the point cloud is explained in detail.

### 3.1. An Overview of LiDAR Data Watermarking

The watermarking methods consist of two weakly coupled tasks: Watermark embedding and watermark extraction.

**Figure 1:**
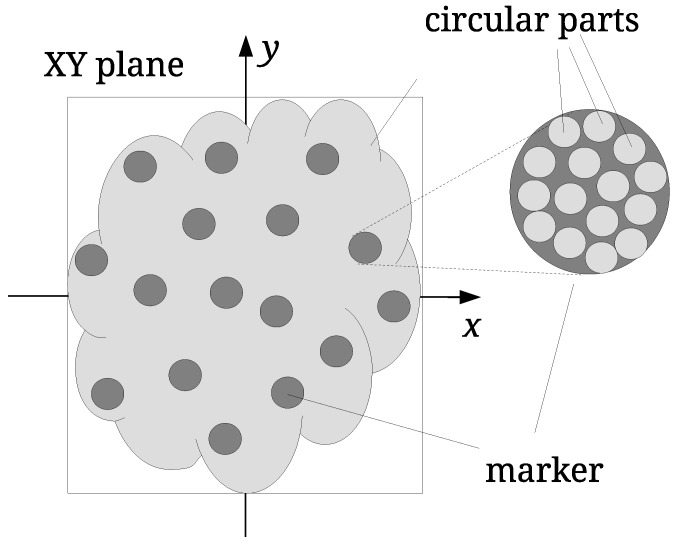
Watermarking of LiDAR point clouds.

- **Watermark embedding.** The watermark embedding defines randomly distributed marker areas, firstly on the XY-plane (see Figure 1). Then, the *X* and *Y* coordinates of the points within these areas are modified slightly as follows: Each marker area is divided further into smaller circular parts. The distances between the centroids of the points within the circular parts and the centres of the circular parts are calculated and used as an input vector to Discrete Cosine Transformation (DCT) [30]. The last DCT-coefficient is modified, and the Inverse Discrete Cosine Transformation (IDCT) is performed. In this way, the modified vector of distances is obtained, and it is used for small disturbances of the centroids. Finally, the coordinates of the LiDAR points are modified slightly to match the new centroid positions (details of the approach are given in [48]).
- **Watermark extraction.** The watermark extraction from the LiDAR point cloud is performed identically to the watermarking, except for the last step [48]. The marker areas are determined first. Then, the distances are calculated, and the vector of the DCT-coefficients is built. Finally, the last DCT-coefficient is checked to determine the value of the embedded watermark bit. The process continues for all marker locations to construct the whole watermark [48].

### 3.2. Point Cloud Watermarking

Contrary to the georeferenced LiDAR points, point clouds, consisting of points scanned from the surfaces of 3D models, can be positioned, scaled, and oriented in many different ways. This is the reason watermarking of such point clouds differs from the LiDAR point clouds’ watermarking. In the continuation of the paper, the original point cloud is denoted as *I*, while the possibly attacked point cloud as $W^{A}$.

Watermark Embedding

**Figure 2:**
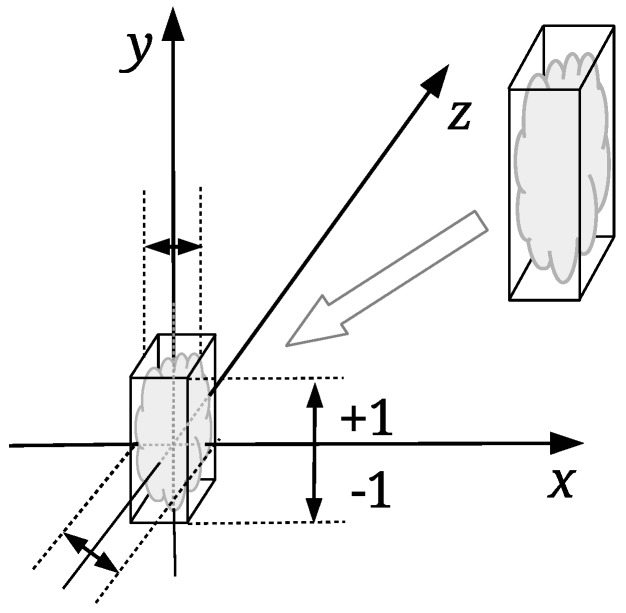
Normalization of input point cloud.

The watermark embedding process should change the coordinates of the points by the same amount regardless of the point cloud size. The input point cloud is normalised first; it is scaled proportionally to map its height between the values −1.0 and 1.0 (see Figure 2). The so-called normalised point cloud $I^{N}$ is obtained from *I* in such a way. The bounding box of *I* is needed only in this step. The normalization of the watermarked, and possible attacked point cloud, is not needed in the process of the point cloud registration. The proposed registration approach is based on the scale ratios between identified triangles of the source and target convex-hulls, as explained in the continuation. The same watermark embedding process is then performed, as described briefly in Section 3.1.

Watermark Extraction

The first step of the watermark extraction from $W^{A}$ is its registration. However, before the registration, $W^{A}$ is cleaned of possible outliers. In our case, the statistical outlier removal algorithm was applied from the Point Cloud Library (PCL) [49,50].

### 3.3. Convex Hull Point Cloud Registration

The convex hull of a set *I* of points in Euclidean space is the smallest convex set that contains *I* [51]. There are various algorithms for constructing convex hulls. In our approach, the Quickhull [46] has been used for *I* and $W^{A}$. Let $H^{W}$ (the source convex-hull) and $H^{I}$ (the target convex-hull) be convex hulls of $W^{A}$ and $I^{N}$, correspondingly. The surface of a 3D convex hull consists of triangles and, therefore, $T^{W}=\left\{t_{i}\right\}$ denotes a set of triangles of $H^{W}$, and $T^{I}=\left\{t_{j}\right\}$ a set of triangles of $H^{I}$. The registration algorithm attempts to find the pairs of corresponding triangles from $T^{W}$ and $T^{I}$, i.e., $t_{i}\approx t_{j}$. If a matching pair of triangles is found, the appropriate scale factor (i.e., the needed affine transformation) can be calculated easily. Unfortunately, various attacks can change $H^{W}$ considerably. Consequently, the number of triangles in both sets can be different, i.e., $|T^{I}\left|\neq \right|T^{W}|$, and the best matching triangles are difficult to identify. Even worse, as explained later, a triangle from $T^{W}$ can have multiple matches with triangles from $T^{I}$. However, if $W^{A}$ and $I^{N}$ are similar to some extent, there should be enough corresponding triangles among $T^{I}$ and $T^{W}$ that the scale and the rigid affine transformation can be estimated. These are then applied to $W^{A}$ for its alignment with $I^{N}$. An Iterative Closest Point algorithm (ICP) was applied for this task  [33,34,35].

Relative Scale Estimation

The areas $A_{i}$ and $A_{j}$ of each triangle $t_{i}$ and $t_{j}$ are calculated first. The triangles of each convex hull are then sorted separately in decreasing order of their areas, and, after that, compared by examining the edge ratios. However, small triangles are not convenient for the comparison, because even the slightest additive noise can change their edge ratios significantly. Thus, 40% of the smallest triangles from $T^{W}$ and 20% of the tiniest triangles from $T^{I}$ are discarded (these percentages were determined experimentally). Next, the lengths of each triangle edges ($d_{1},d_{2}$, and $d_{3}$) are calculated, and arranged in increasing order, i.e., $d_{1}\leq d_{2}\leq d_{3}$. The edges are used to calculate the edge ratios α and β according to Equation (1):

(1) $$\begin{array}{ccc} \alpha & = & d_{1}/d_{3}\\ \beta & = & d_{2}/d_{3} \end{array}$$

The similarity ratios $\sigma_{i,j}$ and $\rho_{i,j}$ are then calculated as follows:

(2) $$\begin{array}{ccc} \sigma_{i,j} & = & \frac{\alpha_{i}}{\alpha_{j}}-1,\;0\leq i<|T^{W}\left|,\;0\leq j<\right|T^{I}|\\ \rho_{i,j} & = & \frac{\beta_{i}}{\beta_{j}}-1,\;0\leq i<|T^{W}\left|,\;0\leq j<\right|T^{I}|. \end{array}$$

Triangle $t_{i}\in T^{W}$ is considered to be similar enough to triangle $t_{j}\in T^{I}$, if $|\sigma_{i,j}\left|<\tau \;\wedge \;\right|\rho_{i,j}|<\tau$, where τ is a given threshold. Arrays of scale factors $S_{i}$ are formed for each $t_{i}$ and matching similar triangles candidates $t_{k}\subseteq T^{I}$ by applying Equation (3):

(3) $$S_{i,k}=\sqrt{\frac{A_{i}}{A_{k}}}.$$

**Figure 3:**
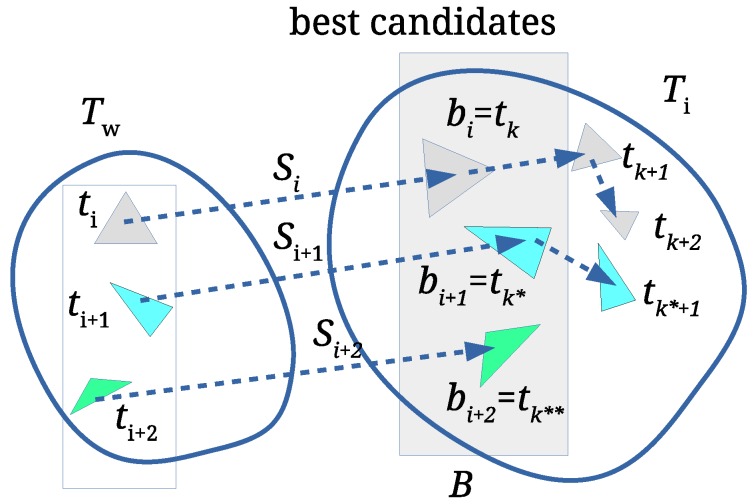
Array of scaling factors $S_{i}$ binds the best candidates ($b_{i},b_{i+1}$, and $b_{i+2}$) for scale estimation.

All arrays $S_{i}$ are then sorted in decreasing order. The first elements of each $S_{i}$ are the best matching triangles and the candidates for the final scale factor estimation (see Figure 3). An array $B=\{\left(b_{i},i_{i},k_{i}\right)\}$ is formed, where $b_{i}$ is the first element of $S_{i}$ (the one with the best scale factor), while $i_{i}$ and $k_{i}$ are indices of corresponding similar triangles $t_{i}\in T^{W}$ and $t_{k}\in T^{I}$, respectively. Array *B* is then sorted in decreasing order according to the $b_{i}$. If $W^{A}$ is obtained by scaling $I^{N}$ with some scale factor *s* and no other attacks occur, then the largest triangles from $H^{W}$ with the similarity ratios $\sigma_{i,j}$ and $\rho_{i,j}$ match with the largest triangles from $H^{I}$ with the same similarity ratios. In such cases, the scale factor *s* would be equal to $b_{i}$, i.e., $s=b_{i}$.

**Figure 4:**
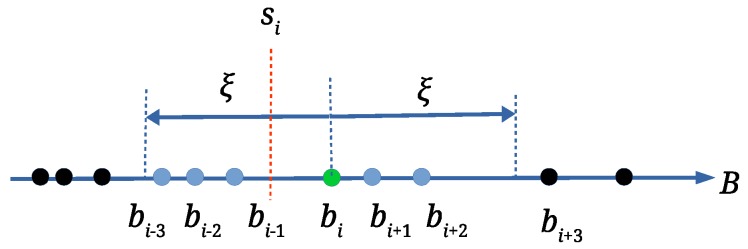
Determining scale factor $s_{i}$ and $f_{i}$; $s_{i}$ in an average of $f_{i}=6$ scale factors in this example.

Unfortunately, attacks cause that $b_{i}$ may have different values. Thus, array *B* is processed in order to determine the most frequent value of the scale factor. An array $H=\{s_{i},f_{i},\Psi_{i}\}$ is established, which is populated by applying Algorithm 1. The scale factor $s_{i}$ is calculated as an average of scale factors within the range $\left[b_{i}-\xi ,b_{i}+\xi \right]$ (ξ is the range threshold, defined experimentally), $\Psi_{i}$ is an array of matching triangle pairs, and $f_{i}$ is the number of scale factors $b_{i}$ within the considered range (see Figure 4). If only a scale attack has occurred, or if there are no attacks at all, at most one scale factor $s_{i}\in H$ with the highest value $f_{i}$ exists. On the other hand, the attacks can cause that the scale factors are dispersed. The wrong scale factors should be isolated, and the correct scale factor should be determined as precisely as possible. Thus, the relatively small range threshold tolerance (ξ=0.0002) is used. As the scale factors $\left(b_{i+1},b_{i+2},\ldots \right)$, that are within range $\left[b_{i}\pm \xi \right]$, are already handled, the next element for processing from *B* is the first scale factor that is outside the range $\left[b_{i}\pm \xi \right]$ (the scale factor $b_{i+3}$ in Figure 4).

**Algorithm 1** Find Most Frequent Scale.**Require:***B*—an array of best scale factors, ξ—the range threshold (default: ξ=0.0002), *n*—the length of the array *B*1:i←02:**while**i<n**do**3:    $s_{i}\leftarrow B.b_{i}$4:    $f_{i}\leftarrow 1$5:    $\Psi_{i}\leftarrow \left\{\left(B.i_{i},B.k_{i}\right)\right\}$6:    j←i−17:    **while** $\left(j>0\;\&\left|B.b_{i}-B.b_{j}\right|<\xi \right)$ **do**8:        $s_{i}\leftarrow \left(s_{i}+B.b_{j}\right)/2$9:        $f_{i}\leftarrow f_{i}+1$10:        $\Psi_{i}\leftarrow \Psi_{i}\cup \left\{\left(B.i_{j},B.k_{j}\right)\right\}\;$11:        j←j−112:    **end while**13:    j←i+114:    **while** $\left(j<n\;\&\left|B.b_{i}-B.b_{j}\right|<\xi \right)$ **do**15:        $s_{i}\leftarrow \left(s_{i}+B.b_{j}\right)/2$16:        $f_{i}\leftarrow f_{i}+1$17:        $\Psi_{i}\leftarrow \Psi_{i}\cup \left\{\left(B.i_{j},B.k_{j}\right)\right\}$18:        j←j+119:    **end while**20:    i←j21:**end while**

**Figure 5:**
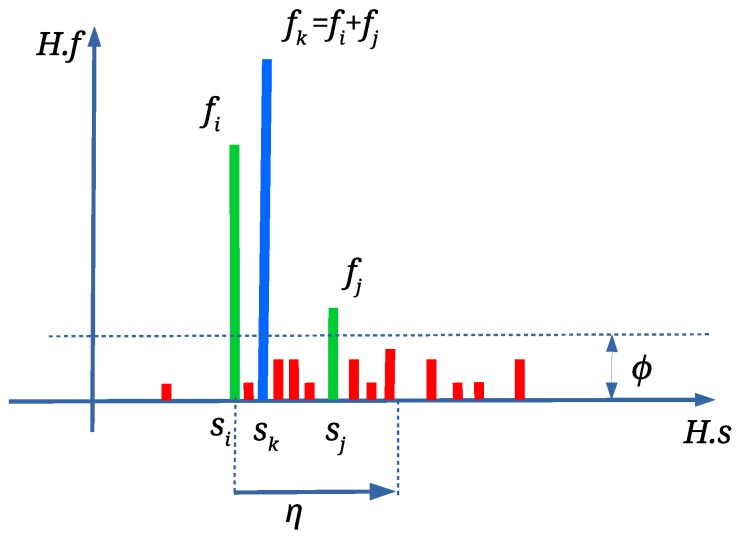
A new $s_{k}$ with frequency $f_{k}=f_{i}+f_{j}$ is obtained from $s_{i}$ and $s_{j}$; scaled factors marked with red colour are discarded.

Any scale factor $s_{i}$ with $f_{i}<\phi$ is discarded in the continuation applying Algorithm 2. ϕ is an occurrence threshold, which was set experimentally to ϕ=3 (see Figure 5). The isolated wrong scale factors are discarded in this way. Depending on the attacks, there can be more than one $s_{i}$ with $f_{i}\geq \phi$. In such case, scale factors are joined, calculating weighted average $s_{k}$ (see Figure 5) with Equation (4). This is performed only if $s_{j}<s_{i}+\eta$ (see Figure 5) (the range threshold η=0.01 was defined experimentally).

(4) $$s_{k}=\frac{s_{i}f_{i}+s_{j}f_{j}}{f_{i}+f_{j}}$$

Triangle pairs $\Psi_{i}$ and $\Psi_{j}$ are joined, too. Finally, *H* is sorted according to $f_{i}$ and the first $s_{i}$ with the highest $f_{i}$ is the best scale estimation.

**Algorithm 2** Scale Factor Discarding And Joining.**Require:***H*—the output from Algorithm 1, η—the range threshold (default: η=0.01, ϕ—the frequency threshold (default: ϕ=3), *n*—is the length of the array *H*)1:**for**i←0ton−2**do**2:    **if** $H.f_{i}>0$ **then**3:        $s\leftarrow H.s_{i}$4:        $f\leftarrow H.f_{i}$5:        $\Psi \leftarrow H.\Psi_{i}$6:        **for** j←0ton−1 **do**7:           **if** $H.f_{j}>\phi \;\&\left|s-H.s_{j}\right|<\eta$ **then**8:               $s\leftarrow \left(sf+H.s_{j}H.f_{j}\right)/\left(f+H.f_{j}\right)$9:               $f\leftarrow f+H.f_{j}$10:               $\Psi \leftarrow \Psi \cup H.\Psi_{j}$11:               $H.f_{j}\leftarrow 0$12:           **end if**13:        **end for**14:        $H.s_{i}\leftarrow s$15:        $H.f_{i}\leftarrow f$16:        $H.\Psi_{i}\leftarrow \Psi$17:    **end if**18:**end for**

Rotation and Translation Estimation

The first $s_{i}$ with the highest $f_{i}$ in the array *H* also contains an array of matching triangles $\Psi_{i}$. They are used for estimation of rotation and translation. Two auxiliary point clouds are built, from the first and the second matching pair of triangles $B.i_{i}$ and $B.k_{i}$. The centres of the triangles are used as the points of these clouds. The source triangles are scaled by $s_{i}$. Because the auxiliary cloud contains considerably fewer points, rotation and translation is performed fast. The rigid transformation matrix (i.e.,rotation and translation) is determined between newly created point clouds by applying the Single Value Decomposition-based alignment estimation [52]. Because the correspondences of the points between these point clouds have been already obtained from the scale estimation process, applying this algorithm is the most efficient solution. The scaling and the rigid transformation are then applied to the watermarked point cloud $W^{A}$. In this way, a better initial alignment hint is assured for the ICP algorithm. The ICP algorithm is applied at the end for the fine alignment of $W^{A}$ to $I^{N}$. As a good initial alignment of the source cloud is achieved, only a few iterations of the ICP are needed.

Extraction of the Watermark Bits

The same steps are performed as in the process of watermark embedding after aligning $W^{A}$ to $I^{N}$ (see Watermark Embedding in Section 3.2 and [48]), except for the last step. The markers are determined; the distances are calculated, and a vector of the DCT coefficients is built. The last DCT coefficient is checked to determine the value of the embedded watermark bit. This process is repeated for all marker locations to reconstruct the whole watermark.

## 4. Experimental Results and Discussions

Stanford 3D models (http://graphics.stanford.edu/data/3Dscanrep/) were used in our experiments (Table 2). Equation (5) was applied to estimate the marker radius $r_{out}$.

(5) $$r_{out}=\sqrt{150\frac{P}{\pi n_{p}}},$$

where $P=d_{x}d_{y}$ is the projected area in plane XY ($d_{x}$ and $d_{y}$ are the sides of the bounding box) (see Figure 1) and $n_{p}$ is the number of points in *I*. Value $n_{p}=150$ was used to achieve that the number of points per marker was in range [150,250]. This guarantees that the watermark can be embedded successfully and sustain a certain level of the distortion caused by various attacks. The 64-bit watermark $63687732303137_{16}$ was applied in all experiments.

The match percentage $m^{p}$ between the inserted watermark *w* and the extracted watermark $w^{*}$ was calculated as:

(6) $$m^{p}=\frac{n^{q}}{M}100\%,$$

where $n^{q}$ is the number of equal bits in both watermarks, and *M* is the size of the watermark in bits. $m^{p}$ indicates the success of watermark extraction from the $W^{I}$ and $W^{A}$. Various experiments were performed to evaluate the proposed approach, and to find how the parameters of the watermarking process affect the presented method.

### 4.1. Setting the Parameters for Watermark Embedding

In this subsection, a short explanation of parameter setting while inserting the watermark into the $W^{I}$ is given (details are in [48]). Table 3 shows the values of the parameters, where $N^{*}$ is the number of markers, *T* is the number of smaller circular areas within the circular marker (i.e.,the number of DCT coefficients), γ is the value of modulating amplitude of the last DCT coefficient that is used to embed a watermark bit, and $d_{max}$ is the maximum allowable distance used to control displacement of the points due to embedding of the watermark.

**Figure 6:**
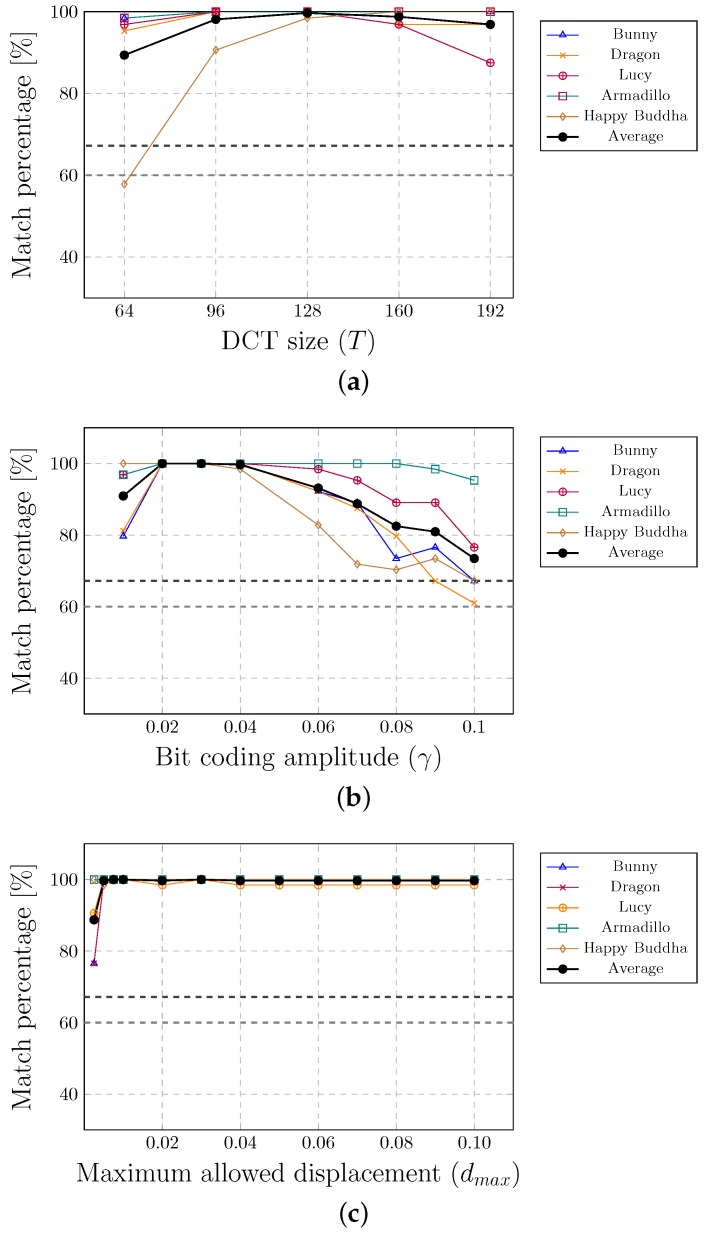
Experimenting with different values of parameters.

Figure 6 shows the results of experiments. The finest results were achieved if the number of smaller circular areas within the marker or the size of the input vector of DCT was between 128 and 160. The best match percentages were obtained when the bit coding amplitude (the amount that the last DCT coefficient was changed) was between 0.02 and 0.04. This parameter has an impact on the level of the point displacement, which is controlled by the parameter $d_{max}$. It was impossible to embed the watermark successfully if this parameter value was too small (below 0.01).

### 4.2. Evaluating the Resistance of the Method Against Different Attack Types

The resistance of the proposed method against different types of attacks is considered in the continuation. Table 4, Table 5, Table 6 and Table 7 show the average ($m_{avg}^{p}$) and the minimum match percentage ($m_{min}^{p}$), together with the number of successful watermark extractions ($n^{e}$) above threshold $m_{th}^{p}$. The $m_{th}^{p}$ value is set to 67.19% as determined in [48]. The last column of Table 4, Table 5, Table 6 and Table 7 represents the average $m_{avg}^{p}$ and the minimum $m_{min}^{p}$ match percentage of all test cases (marked with ∗), while $n^{e}$ is the total number of successful watermark extractions (marked with †).

#### 4.2.1. Affine Transformation Attacks

**Figure 7:**
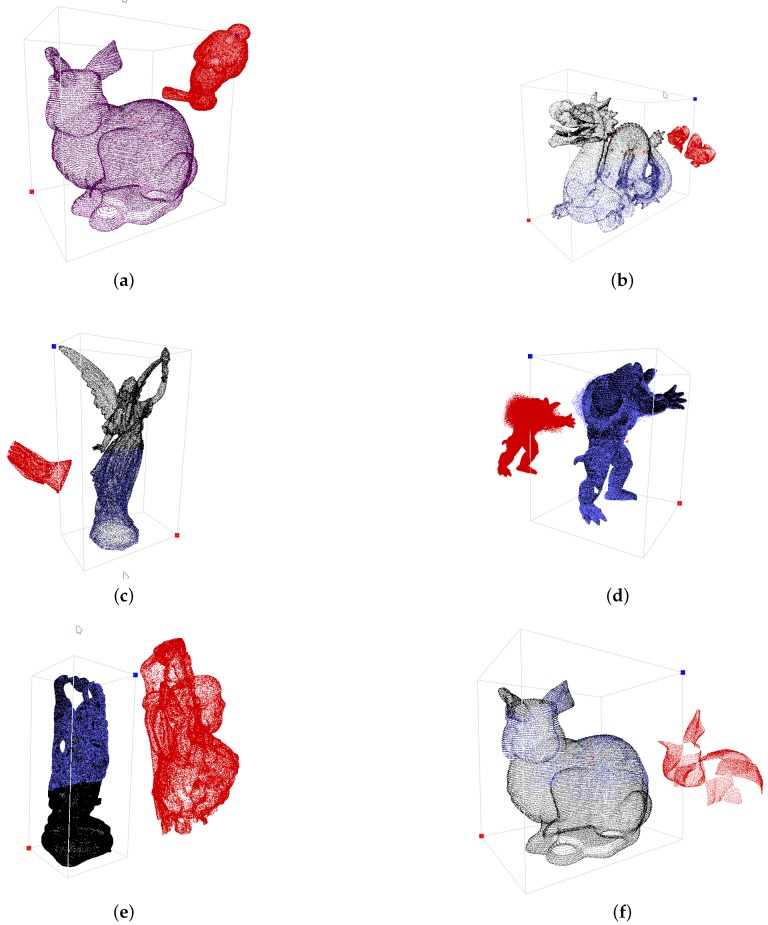
Various examples of combined attacks: (**a**) Affine transformation ($m^{p}=100\%$). (**b**) Affine transformation and cropping ($m^{p}=95.31\%$). (**c**) Affine transformation, cropping and random removal ($m^{p}=93.75\%$). (**d**) Affine transformation and local noise ($m^{p}=96.88\%$). (**e**) Affine transformation and cropping with slantwise cut ($m^{p}=87.50\%$). (**f**) Affine transformation and complex cropping ($m^{p}=93.51\%$).

The main advantage of the PCA-based approaches is robustness against affine transformation attacks (see Table 1 in Section 2). Therefore, the proposed method was faced firstly with this type of attack. One-hundred random test cases were performed for each point cloud. The scaling factor was chosen randomly between 0.1 and 4.0, the translation in each coordinate direction between −2.0 and 2.0, and the rotation around each axis between 0.0 and 2π radians. The results of individual and combined affine transformation attacks are given in Table 4. The watermark was unable to extract only in 3 out of 500 cases. Although the majority of test cases succeeded, we have checked the failed cases. It turns out that the orientation of $W^{A}$ was inappropriate for the ICT algorithm in all three cases. A slight rotation of $W^{A}$ remedied the problem. Figure 7a shows an example of the combined affine transformation attacks, where $W^{A}$ is in the red colour. The point cloud was aligned completely with the original one and, the match percentage $m^{p}=100\%$.

#### 4.2.2. Cropping Attacks

**Figure 8:**
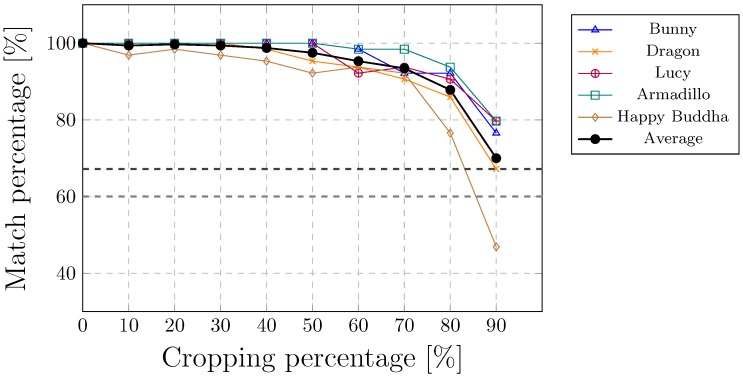
Cropping top attack.

PCA-based methods cannot handle cropping attacks because of calculating the point cloud centroid. Even the smallest cropping may, therefore, have a huge impact on the position of a centroid. On the contrary, the proposed method copes well with the cropping attacks. In our case, $W^{A}$ was cropped from the top by various amounts as shown in Figure 8. The watermark was extracted, even when $W^{A}$ was cropped to 80% of the points. A combination of affine transformations and cropping attacks was the next experiment. The amount of croppings were changing randomly between 0% and 50%. The majority of the cases were solved successfully, as shown in Table 5. Only 9 out of 500 test cases failed, i.e.,the watermark was not extracted. Figure 7b shows an example where 60% of $W^{A}$ points was cropped (the attacked 3D point cloud is plotted in red, the aligned model in blue, while the original points cloud is displayed in black). Another example of a cropping attack is presented in Figure 7e, where the bottom of $W^{T}$ was cropped with a slantwise cut at approximately 50%. The watermark was extracted successfully in both cases.

#### 4.2.3. Random Removal Attacks

**Figure 9:**
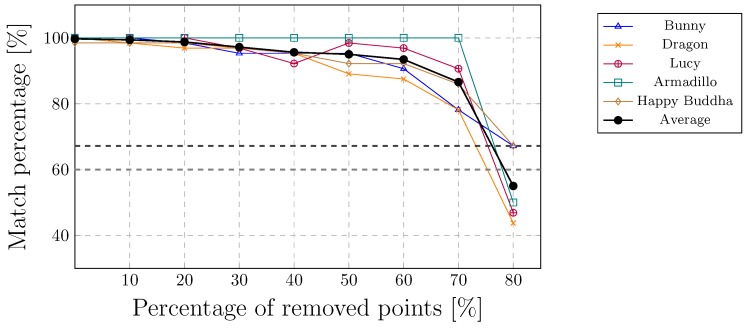
Random removal of the points.

The cluster-based approach, developed by Agarwal and Prabhakaran [27], constructs a cluster-tree using the nearest neighbour heuristic. Thus, their method is vulnerable to random removal attacks that have an impact on the cluster construction. Figure 9 shows that the proposed method can extract the watermark, even up to 70% of randomly removed points.

The combined cropping and random removal attacks were performed (Table 6) in the next experiments. All 500 test cases succeeded. The amount of cropping and the percentage of removed points were changed randomly between 0% and 50%. Table 6 also shows the results of the combined affine transformation, cropping, and random removal attacks. Our method extracted the watermarks in 488 out of the 500 test cases. Figure 7c shows an example where 50% of points were cropped, and then 50% of the remaining points were removed randomly. The method was successful in this case. Another example of a cropping attack is presented in Figure 7f, where even more complex cropping was performed (large portions of the model are removed from the top, while the bottom of the model is removed completely).

#### 4.2.4. Noise Attacks

**Figure 10:**
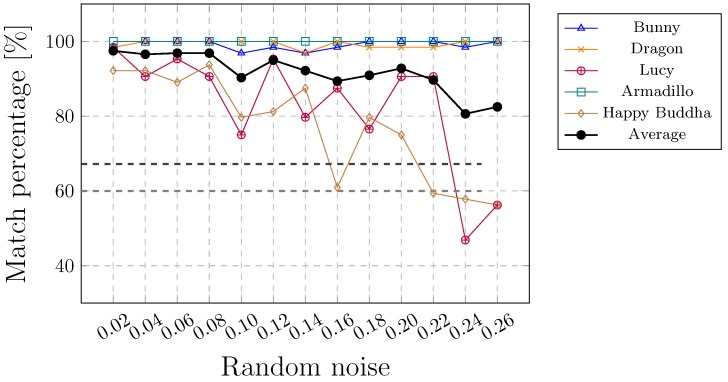
Local noise attack.

**Figure 11:**
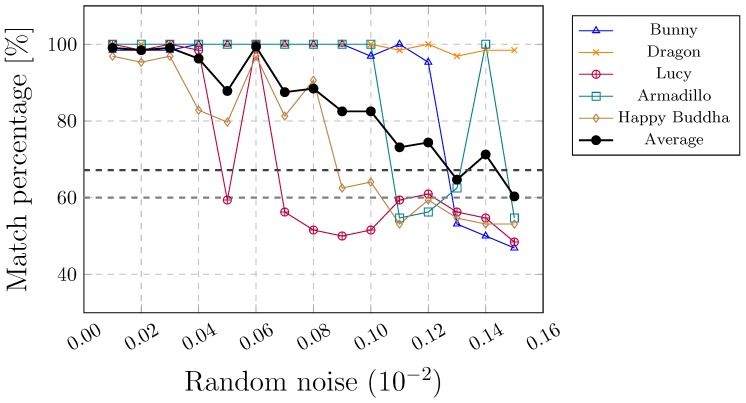
Global noise attack.

This kind of attack is considered to be the most difficult. As the watermark is embedded by changing the coordinates of the points, any further alteration can damage the embedded watermark regardless of the watermarking approach. Thus, none of the existing methods can resist this kind of attack adequately. Indeed, the watermark can be protected only to a certain extent. A local noise attack is less vulnerable than a global noise attack. The watermark can resist a higher amount of locally, rather than globally, added noise (see Figure 10 and Figure 11). This can also be seen in Table 7 that summarises the results of the combined attacks. Figure 11d shows an example where the noise between 0.00 and 0.18 (according to the height of the 3D model, normalised between −1 and 1) was added randomly in the upper part of $W^{A}$. The noise may have an impact on the convex hull. It can change the edges of triangles in such a way that the correspondence between triangles $t_{i}$ and $t_{j}$ cannot be found.

## 5. Conclusions

A new method for 3D point cloud watermarking is considered in this work. The scale estimation and the registration of a possibly attacked point cloud with the source point cloud are done through the use of a 3D convex hull. The method consists of the following three steps:

- Constructing the convex hulls of watermarked and original point clouds;
- Matching the triangles of both convex hulls to determine the affine transformation between them;
- Performing point cloud registration by applying the obtained affine transformation and the Iterative Closest Point registration algorithm.

Extensive testing was done of the proposed method. It was confirmed that the method can handle cropping attacks, which cannot be tackled by the existing PCA-based approaches. The main benefit of these approaches is their resistance to affine transformation attacks. It was shown that the proposed method copes with this type of attack equally well. The method is also successful with other attacks, such as random removal attacks, affine transformation attacks, and combinations of all the mentioned attacks. The most devastating would be an attack with noise, because it cancels out the modifications in 3D point positions, made during the process of the watermark embedding. However, this issue is common with all known 3D point clouds watermarking methods.

## Figures and Tables

**Table 1:** The watermark robustness (h—high, l—low or non-robust (marked with ×)) against various attacks.

Attack	Approach							
	Our	PCA-Based			Other			
		[24]	[26]	[25]	[27]	[29]	[32]	[31]
affine-transformation	h	h	h	h	h	×	l	l
cropping	h	×	×	×	l	*l*	×	×
random removal	h	l	l	l	l	×	×	×
local noise	h ^†^	h ^†^	l	h ^†^	h ^†^	l	l	l
global noise	l	l	l	l	l	l	×	×

^†^ This depends strongly on the level of locally added noise.

**Table 2:** 3D point clouds used in experiments.

File	3D Model	Number of Points $n_{p}$	Number of Convex Hull Triangles	$r_{\mathrm{out}}$
F1	Bunny	35,947	2064	0.073212
F2	Dragon	50,000	752	0.073595
F3	Lucy	50,002	624	0.047158
F4	Armadillo	172,974	1668	0.030441
F5	Happy Buddha	543,652	3734	0.012011

**Table 3:** The values of parameters.

Parameter	Range	Default Value
$N^{*}$	-	4000
*T*	64–192	128
γ	0.02–0.10	0.04
$d_{max}$	0.0025–0.2000	0.0040

**Table 4:** Affine transformation attacks.

File	F1	F2	F3	F4	F5	Avg/Min ∗/Total †
**Translate attack**						
$m_{avg}^{p}$ (%)	99.98	100.00	100.00	100.00	98.27	98.27
$m_{min}^{p}$ (%)	98.44	100.00	100.00	100.00	93.75	93.75 ∗
$n^{e}$	100	100	100	100	100	500 †
**Scale attack**						
$m_{avg}^{p}$ (%)	99.91	100.00	100.00	100.00	98.91	98.27
$m_{min}^{p}$ (%)	98.44	100.00	100.00	100.00	93.75	93.75 ∗
$n^{e}$	100	100	100	100	100	500 †
**Rotate attack**						
$m_{avg}^{p}$ (%)	99.38	99.91	97.27	100.00	95.85	98.48
$m_{min}^{p}$ (%)	98.44	98.44	56.25	100.00	71.88	56.25 ∗
$n^{e}$	100	100	98	100	100	498 †
**Combined affine transformation attacks**						
$m_{avg}^{p}$ (%)	99.33	99.89	97.45	100.00	91.49	98.23
$m_{min}^{p}$ (%)	98.44	98.44	62.55	100.00	78.12	62.55 ∗
$n^{e}$	100	100	99	100	100	499 †

**Table 5:** Combined affine transformation and cropping attacks.

File	F1	F2	F3	F4	F5	Avg/Min ∗/Total †
$m_{avg}^{p}$ (%)	99.56	98.08	95.14	100.00	87.44	96.04
$m_{min}^{p}$ (%)	96.88	89.06	71.88	100.00	53.12	53.12 ∗
$n^{e}$	100	100	100	100	91	491 †

**Table 6:** Combined attacks with a random removal of points.

File	F1	F2	F3	F4	F5	Avg/Min ∗/Total †
**Combined cropping and random removal attacks**						
$m_{avg}^{p}$ (%)	96.52	95.31	98.34	100.00	92.17	96.47
$m_{min}^{p}$ (%)	85.94	81.25	87.50	100.00	71.88	71.88 ∗
$n^{e}$	100	100	100	100	100	500 †
**Combined affine trans., cropping and random removal attacks**						
$m_{avg}^{p}$ (%)	97.33	94.20	91.75	99.98	85.47	93.75
$m_{min}^{p}$ (%)	85.94	81.25	98.44	54.69	54.96	93.75 ∗
$n^{e}$	100	100	99	100	89	488 †

**Table 7:** Combined attacks with noise added locally between 0.02 and 0.26 and a maximum noise added globally between 0.0001 and 0.0002.

File	F1	F2	F3	F4	F5	Avg/Min ∗/Total †
**Combined affine trans., cropping, random removal and local noise attacks**						
$m_{avg}^{p}$ (%)	96.76	93.066	77.28	91.86	66.89	85.27
$m_{min}^{p}$ (%)	85.94	76.56	40.62	48.44	45.31	45.31 ∗
$n^{e}$	100	100	70	81	41	395 †
**Combined affine trans., cropping, random removal and global noise attacks**						
$m_{avg}^{p}$ (%)	97.33	94.09	73.86	94.06	80.59	87.99
$m_{min}^{p}$ (%)	87.50	57.81	35.94	43.75	53.12	35.94 ∗
$n^{e}$	100	99	55	88	78	420 †

## Abbreviations

The following abbreviations are used in this manuscript:

PCA	Principal component analysis
DCT	Discrete cosine transformation
IDCT	Inverse discrete cosine transformation
LiDAR	Light Detection And Ranging
ICP	Iterative closest point algorithm
RANSAC	Random sample consensus
PCL	Point cloud library
